# A systematic review of the effectiveness of docetaxel and mitoxantrone for the treatment of metastatic hormone-refractory prostate cancer

**DOI:** 10.1038/sj.bjc.6603287

**Published:** 2006-08-01

**Authors:** R Collins, R Trowman, G Norman, K Light, A Birtle, E Fenwick, S Palmer, R Riemsma

**Affiliations:** 1 Centre for Reviews and Dissemination, University of York, Heslington, York YO10 5DD, UK; 2 Rosemere Cancer Centre, Royal Preston Hospital, Sharoe Green Lane North, Fulwood, Preston PR2 9HT, UK; 3 Public Health & Health Policy, Division of Community Based Sciences, University of Glasgow, 1 Lilybank Gardens, Glasgow G12 8RZ, UK; 4 Centre for Health Economics, University of York, Heslington, York YO10 5DD, UK; 5 Kleijnen Systematic Reviews Ltd, Westminster Business Centre, 10 Great North Way, Nether Poppleton, York YO26 6RB, UK

**Keywords:** hormone-refractory prostate cancer, docetaxel, mitoxantrone, systematic review

## Abstract

A systematic review was performed to evaluate the clinical effectiveness of docetaxel in combination with prednisolone (docetaxel is licensed in the UK for use in combination with prednisone or prednisolone for the treatment of patients with metastatic hormone-refractory prostate cancer. Prednisone is not used in the UK, but it is reasonable to use docetaxel plus prednisone data in this review of docetaxel plus prednisolone) for the treatment of metastatic hormone-refractory prostate cancer. A scoping search identified a trial of docetaxel plus prednisone *vs* mitoxantrone plus prednisone, but did not identify any trials comparing docetaxel plus prednisolone/prednisone with any other treatments. Therefore, we considered additional indirect evidence that would enable a comparison of docetaxel plus prednisolone/prednisone with other chemotherapy regimens and active supportive care. Systematic searching (upto April 2005) identified seven randomised controlled trials. One large well-conducted trial assessed docetaxel plus prednisone *vs* mitoxantrone plus prednisone; this showed statistically significant improvements with 3-weekly docetaxel in terms of overall survival, quality of life, pain response and PSA decline. Two other chemotherapy regimens that included docetaxel with estramustine also showed improved outcomes in comparison with mitoxantrone plus prednisone. Three trials that compared mitoxantrone plus corticosteroids with corticosteroids alone were identified and their results for overall survival combined, which showed very little difference between the two groups. The addition of clodronate to mitoxantrone plus prednisone showed no significant differences in comparison with mitoxantrone plus prednisone alone. The evidence suggests that chemotherapy regimens containing 3-weekly docetaxel are superior to mitoxantrone or corticosteroids alone.

Prostate cancer is the most common cancer among men in the UK (excluding nonmelanoma skin cancer), the lifetime risk for being diagnosed with prostate cancer is one in 13 (Cancer Research UK, 2004). The majority of patients are diagnosed with early disease and have a good prognosis. However, approximately 22% of cases will be diagnosed with advanced or metastatic disease (National Institute for Clinical Excellence, 2002), with an additional 25% developing metastases throughout the course of the disease (Muthuramalingam *et al*, 2004). The majority of prostate cancers initially respond to hormone therapy, with a median response duration in patients with metastatic disease of around 18 months (Eisenberger *et al*, 1998). However, in most patients with metastatic disease, the cancer will become resistant to hormonal treatment and will progress to metastatic hormone-refractory prostate cancer (mHRPC). Metastatic hormone-refractory prostate cancer is defined as either biochemically or clinically progressive metastatic disease despite castrate serum levels of testosterone (Muthuramalingam *et al*, 2004). The prognosis for mHRPC is poor, and survival in men with symptomatic metastases is not expected to exceed between 9 and 12 months, once they have failed first-line hormonal therapy (Petrylak, 2002).

Treatment for mHRPC is palliative. Options include second-line hormonal therapy, chemotherapy and active supportive care, and patients may receive a combination of palliative treatments. Current advice issued by the National Institute for Health and Clinical Excellence (NICE) states that chemotherapy should be considered and trials of chemotherapy supported (National Institute for Clinical Excellence, 2002). The taxane docetaxel (Taxotere®, Sanofi-Aventis, Guildford, UK) has recently been licensed for the treatment of mHRPC in the UK, in combination with prednisolone. (Docetaxel is licensed for use in combination with prednisone or prednisolone for the treatment of patients with mHRPC. Prednisone is not used in the UK, but it is reasonable to use docetaxel plus prednisone data in this review of docetaxel plus prednisolone.)

The Centre for Reviews and Dissemination (CRD) and the Centre for Health Economics were commissioned to conduct a systematic review on behalf of NICE of the clinical and cost-effectiveness of docetaxel for the treatment of mHRPC. This paper presents the systematic review of the effectiveness evidence. A full technical report is available, which also presents the results of the economic evaluation that was conducted alongside the systematic review (Collins *et al*, 2006).

The objective of the systematic review was to evaluate the clinical effectiveness of docetaxel in combination with prednisolone *vs* other chemotherapy regimens, active supportive care (which may include radiotherapy, corticosteroids and analgesics) or placebo for the treatment of mHRPC.

## MATERIALS AND METHODS

A scoping search was conducted which identified a trial of docetaxel plus prednisone *vs* mitoxantrone (Novantrone®, Wyeth, Maidenhead, UK) plus prednisone (Tannock *et al*, 2004). The scoping search did not identify any trials comparing docetaxel plus prednisolone/prednisone with any of the other relevant treatments. However, trials comparing mitoxantrone with other chemotherapies and corticosteroids (used as active supportive care) were identified. Therefore, in order to allow for a comparison between docetaxel and other relevant treatments (albeit indirect), the clinical effectiveness of mitoxantrone *vs* other relevant treatments was also reviewed. Mitoxantrone is not licensed for the treatment of mHRPC in the UK. However, it is licensed in combination with corticosteroids for mHRPC in the USA and is widely given for metastatic prostate cancer in the UK. In order to be inclusive, we assessed mitoxantrone in combination with any form of corticosteroid.

Twenty-one electronic resources (MEDLINE, EMBASE, Cochrane Central Register of Controlled Trials (CENTRAL), the Cochrane Database of Systematic Reviews (CDSR), National Research Register (NRR), Health Technology Assessment Database (HTA), NHS Economic Evaluation Database (NHS EED), Database of Abstracts of Reviews of Effects (DARE), CINAHL, Health Management Information Consortium (HMIC), ISI Science and Technology Proceedings, Social Science Citation Index, Index to Theses, SIGLE, Inside Conferences, BIOSIS Previews, Current Controlled Trials, ClinicalTrials.gov, International Cancer Research Portfolio (ICRP), National Cancer Institute Clinical Trials PDQ, American Society of Clinical Oncology) were searched from inception to April 2005. Index and free text terms were used to search for prostate cancer and these terms were combined with index and free text terms for docetaxel (including generic and trade names). Separate searches were performed to combine the prostate cancer terms with index and free text terms for mitoxantrone. Where possible, subject index terms were used to exclude animal studies. No language, date or study-type restrictions were applied. The reference lists of included and background papers were also searched for additional relevant studies.

Published and unpublished randomised controlled trials (RCTs) that compared docetaxel in combination with prednisolone/prednisone (as per its licensed indication) with any chemotherapy regimen or active supportive care or placebo in men with mHRPC were included. Randomised controlled trials that assessed mitoxantrone in combination with a corticosteroid (not licensed for mHRPC in the UK, but licensed in combination with corticosteroids in the USA) compared with any chemotherapy regimen or active supportive care or placebo in men with mHRPC were also eligible for inclusion. Data on the following outcomes were included: overall survival, progression-free survival, response rate (including complete and partial response), PSA decline, adverse effects of treatment, pain and health-related quality of life.

Studies that were reported in abstract form only, where no further information was available, and foreign language papers were excluded. Where multiple publications of the same study were identified, data were extracted and reported as a single study. The quality of RCTs was assessed according to criteria based on CRD Report No. 4 (Centre for Reviews and Dissemination, 2001). Each stage of the review process was conducted by two reviewers, with disagreements resolved by consensus or referral to a third reviewer. A narrative synthesis is presented and, where appropriate, outcomes were synthesised using formal analytic approaches. Full details of the review methods, including the search strategies, are described in the full technical report (Collins *et al*, 2006).

## RESULTS

**Figure 1:**
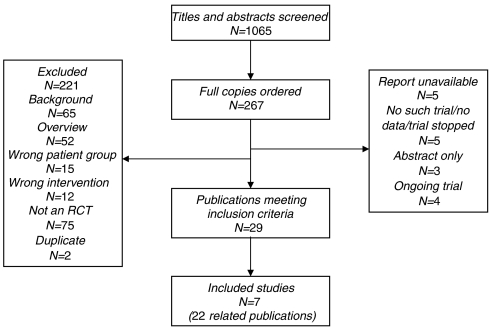
Process of study selection.

A total of 1065 titles and abstracts were screened for inclusion in the review and 267 records were ordered as full papers. Seven RCTs were identified that met our inclusion criteria. The process of study selection is shown in Figure 1.

Of the seven RCTs included, three used docetaxel compared with mitoxantrone plus prednisone (Petrylak *et al*, 2004; Tannock *et al*, 2004; Oudard *et al*, 2005), three used mitoxantrone plus a corticosteroid compared with a corticosteroid (used as active supportive care) (Tannock *et al*, 1996; Kantoff *et al*, 1999; Berry *et al*, 2002) and one used mitoxantrone plus prednisone compared with mitoxantrone plus prednisone plus clodronate (Ernst *et al*, 2003). There were no trials comparing docetaxel plus prednisolone/prednisone or mitoxantrone plus a corticosteroid with other types of chemotherapy or active supportive care. A summary of the pattern of comparisons for the included RCTs is presented in Table 1 and detailed characteristics of the included RCTs are presented in Table 2.

We identified one large well-conducted RCT that assessed the intervention under consideration: docetaxel plus prednisone; this was in comparison with mitoxantrone plus prednisone (TAX 327 trial) (Tannock *et al*, 2004). No other RCTs were found that assessed the clinical effectiveness of docetaxel plus prednisone. The RCT included 1006 men with mHRPC and stable levels of pain. Patients were randomised to receive 3-weekly docetaxel plus prednisone, weekly docetaxel plus prednisone or mitoxantrone plus prednisone. The results of this RCT showed statistically significant improvements with 3-weekly docetaxel plus prednisone compared with mitoxantrone plus prednisone in terms of overall survival (HR=0.76 (95% CI: 0.62, 0.94)), quality of life response (RR=1.67 (95% CI: 1.14, 2.45)), pain response (RR=1.58 (95% CI: 1.1, 2.27)) and PSA decline (defined as a reduction in serum PSA levels of 50% from baseline) (RR=1.41 (95% CI: 1.14, 1.73)). Response rate (defined as objective tumour response assessed using World Health Organisation criteria) was higher for the 3-weekly docetaxel plus prednisone group than the mitoxantrone plus prednisone group, but this difference was not statistically significant. The improved outcomes for docetaxel plus prednisone were associated with more grade 3-4 adverse events. Progression-free survival was not assessed in this RCT. There were also statistically significant improvements with weekly docetaxel plus prednisone compared with mitoxantrone plus prednisone in terms of quality of life response (RR=1.75 (95% CI: 1.20, 2.56)) and PSA decline (RR=1.5 (95% CI: 1.22, 1.84)), but not for any of the other outcomes assessed.

We found three RCTs comparing mitoxantrone plus prednisone with another chemotherapy regimen: one small RCT compared mitoxantrone plus prednisone with 3-weekly docetaxel plus prednisone plus estramustine and docetaxel twice every 3 weeks plus prednisone plus estramustine in 130 men with mHRPC (Oudard *et al*, 2005); one RCT compared mitoxantrone plus prednisone with 3-weekly docetaxel plus estramustine in 770 men with mHRPC (Petrylak *et al*, 2004); and one double-blind RCT compared mitoxantrone plus prednisone plus placebo with mitoxantrone plus prednisone plus clodronate in 227 men with mHRPC and stable levels of analgesic use (Ernst *et al*, 2003). Overall survival and progression-free survival were statistically significantly improved with docetaxel plus estramustine compared with mitoxantrone plus prednisone, response rate was statistically significantly improved with docetaxel plus prednisone plus estramustine compared with mitoxantrone plus prednisone and PSA decline was statistically significantly improved for both regimens containing docetaxel compared with mitoxantrone plus prednisone. Docetaxel plus estramustine was associated with more adverse events, compared with mitoxantrone plus prednisone. No significant differences were found between mitoxantrone plus prednisone plus clodronate and mitoxantrone plus prednisone without clodronate.

In addition, we found three RCTs that compared mitoxantrone plus a corticosteroid with a corticosteroid alone (used as active supportive care). Two of these compared mitoxantrone plus prednisone with prednisone (5 mg twice daily) (Tannock *et al*, 1996; Berry *et al*, 2002), whereas one compared mitoxantrone plus hydrocortisone with hydrocortisone (40 mg given in two divided doses daily) (Kantoff *et al*, 1999). One of the RCTs included 120 men with asymptomatic mHRPC (Berry *et al*, 2002); another included 161 men with symptomatic mHRPC, with symptoms including pain and disease progression (Tannock *et al*, 1996); while the third study included all men with progressive mHRPC (*n*=242) (Kantoff *et al*, 1999). One RCT allowed patients to crossover during the trial, this resulted in 50 out of 81 patients randomised to prednisone to receive additional mitoxantrone (Tannock *et al*, 1996); the other two RCTs did not allow crossovers.

The combined result of these three RCTs showed no significant difference between mitoxantrone plus a corticosteroid compared with a corticosteroid alone in terms of overall survival (HR=0.99 (95% CI: 0.82, 1.20)). This result was exactly the same using fixed effect and random effects approaches. Other outcomes could not be pooled because they were measured differently in the three RCTs. However, in the two studies that measured health-related quality of life and pain response, the mitoxantrone groups had statistically significant improvements compared with the corticosteroid groups for several of the quality of life and pain items assessed (Tannock *et al*, 1996; Kantoff *et al*, 1999). High losses to follow-up for these outcomes dictate that these results should be interpreted cautiously.

In addition to the seven RCTs, four ongoing studies were also identified. However, no further details of the studies were obtainable from the trialists. The interventions that were assessed in these trials were as follows: docetaxel plus prednisone plus placebo *vs* docetaxel plus prednisone plus bevacizumab (Kelly, 2005), docetaxel plus prednisone *vs* GVAX® prostate cancer vaccine (Cell Genesys), docetaxel plus prednisolone *vs* docetaxel plus prednisolone plus zoledronic acid *vs* docetaxel plus prednisolone plus strontium-89 *vs* docetaxel plus prednisolone plus zoledronic acid plus strontium-89 (Trapeze trial) (James) and mitoxantrone *vs* paclitaxel plus carboplatin (Cabrespine *et al*).

## DISCUSSION

### Key findings

The results of the only identified RCT to assess the intervention under consideration (docetaxel plus prednisone) showed statistically significant improvements compared with mitoxantrone plus prednisone in terms of overall survival, quality of life, pain response and PSA decline (when given in 3-weekly doses). The improved outcomes for docetaxel plus prednisone were associated with more grade 3–4 adverse events; however, this had no detrimental effect on quality of life, which was significantly improved. There were statistically significant improvements in quality of life and PSA decline for patients receiving weekly docetaxel, however, survival and pain response were not statistically significantly improved. Two other chemotherapy regimens that included docetaxel: docetaxel plus estramustine and docetaxel plus prednisone plus estramustine, also showed improved outcomes in comparison with mitoxantrone plus prednisone in terms of overall survival, progression-free survival, response rate and PSA decline. However, docetaxel in combination with estramustine was associated with more adverse events. It should be noted that these trials included patients with a Karnofsky performance status score of at least 60%, or Eastern Cooperative Oncology Group or Southwest Oncology Group performance status score of 0–2; therefore, the results can only be generalised to patients with a similar performance status.

Three trials that compared mitoxantrone plus a corticosteroid with a corticosteroid alone were identified and their results for overall survival were combined, which showed very little difference between the two groups. The addition of clodronate to mitoxantrone plus prednisone showed no significant differences in comparison with mitoxantrone plus prednisone alone.

### Limitations

The review was limited by the lack of trials assessing the intervention under consideration: docetaxel plus prednisone. As docetaxel plus prednisone has only been directly compared with mitoxantrone plus prednisone, we considered additional evidence that would enable a comparison of docetaxel plus prednisone with other chemotherapy-based treatments and active supportive care. The only form of active supportive care for which evidence was available was corticosteroids. Therefore, no conclusions can be drawn regarding the comparative effectiveness of docetaxel to other types of active supportive care.

The small number of studies identified prevented the assessment of publication bias using funnel plots or the Egger test (Egger *et al*, 1997). However, the risk is likely to be low, considering the attempts to locate unpublished data, such as searching grey literature and trials databases.

The three pooled RCTs that compared mitoxantrone plus a corticosteroid with a corticosteroid alone differed in terms of the patient population and study methodology, and in terms of whether patients were allowed to crossover between treatment groups. The difference in disease severity between patients included in the RCTs may have affected the results; mitoxantrone was more effective in the RCT of patients with symptoms of pain and least effective in the RCT that only included asymptomatic patients. However, the patients can be regarded as a relatively homogeneous subset of patients with progressive mHRPC that were healthy enough to receive chemotherapy. Including crossovers in intention-to-treat analyses can result in ‘dilution’ of the true effects of a treatment, as patients are analysed as randomised. However, in this case the study that allowed crossovers had a stronger treatment effect in terms of overall survival in favour of mitoxantrone plus prednisone than the two studies that did not allow crossovers.

### Implications for clinical practice

Docetaxel chemotherapy is the first agent to offer a survival advantage, albeit modest, in patients with mHRPC. Of more relevance is the demonstrable improvement in overall quality of life, seen in almost twice the number of patients as those treated with mitoxantrone and prednisone. This improvement takes into account any side effects encountered owing to the chemotherapy. Pain, which for many patients is the most debilitating symptom and can be difficult to manage, was reduced in one-third of patients treated with docetaxel. It is clear that docetaxel chemotherapy is not without toxicity, and that the patients who will gain from treatment are those with good performance status (Karnofsky score of 60% or more). Careful patient selection by the oncologist therefore remains paramount.

### CONCLUSIONS

The limited evidence suggests that 3-weekly docetaxel plus prednisone is superior to mitoxantrone plus prednisone in terms of overall survival, quality of life, pain and PSA decline, and that mitoxantrone plus a corticosteroid does not improve survival compared with a corticosteroid alone. At the time of this assessment, there were ongoing trials of docetaxel as the standard arm in combination with other therapies.

## Figures and Tables

**Table 1:** Treatment comparisons

	**Treatment comparisons**					
**Trial**	**D^a^+P**	**D^a^+P+E**	**D+E**	**M+C**	**M+C+Clo**	**C**
TAX 327	✓			✓(M+P)		
Oudard *et al* (2005)		✓		✓(M+P)		
SWOG 9916			✓	✓(M+P)		
Berry *et al* (2002)				✓(M+P)		✓(P)
CCI-NOV22				✓(M+P)		✓(P)
CALGB 9182				✓(M+H)		✓(H)
Ernst *et al* (2003)				✓(M+P)	✓	

D=docetaxel; P=prednisone/prednisolone; E=estramustine; M=mitoxantrone; C=corticosteroid (either prednisone or hydrocortisone); Clo=clodronate; H=hydrocortisone.

a Evaluated at two different dosages.

**Table 2:** Summary of included RCTs

**Study details**	**Results**
**TAX 327 (Sanofi-Aventis)** Tannock *et al* (2004)	**Mortality:** Intervention A *vs* control HR=0.76 (95% CI: 0.62, 0.94). Intervention B *vs* control HR=0.91 (95% CI: 0.75, 1.11).
**Study design:** Multicentre, stratified open-label RCT.	**Progression-free survival:** Not reported.
**Participants:** 1006 men with metastatic prostate cancer, with disease progression during hormonal therapy. Patients were required to have stable levels of pain for at least 7 days before randomisation.	**Response rate:** Intervention A *vs* control RR=1.65 (95% CI: 0.78, 3.48). Intervention B *vs* control RR=1.12 (95% CI: 0.49, 2.56).
**Intervention A (*n*=335):** Docetaxel (75 mg m^−2^ on day 1 every 21 days)+prednisone or prednisolone (5 mg orally twice daily from day 1) *vs*	**Quality of life response:** Intervention A *vs* control RR=1.67 (95% CI: 1.14, 2.45). Intervention B *vs* control RR=1.75 (95% CI: 1.20, 2.56).
**Intervention B (*n*=334):** Docetaxel (30 mg m^−2^ on days 1, 8, 15, 22 and 29 in a 6-week cycle)+prednisone or prednisolone (as above) *vs*	**Pain response:** Intervention A *vs* control RR=1.58 (95% CI: 1.1, 2.27). Intervention B *vs* control RR=1.40 (95% CI: 0.96, 2.03).
**Control (*n*=337):** Mitoxantrone (12 mg m^−2^ on day 1 every 21 days)+prednisone or prednisolone (as above).	**PSA decline:** Intervention A *vs* control RR=1.41 (95% CI: 1.14, 1.73). Intervention B *vs* control RR=1.5 (95% CI: 1.22, 1.84).
	**Grade 3/4 adverse events:** Intervention A=46%, intervention B=43%, control=35%.
	
Oudard *et al* (2005)	
**Study design:** Multicentre, stratified open-label RCT.	**Mortality:** Intervention A *vs* control HR=0.94 (95% CI: 0.29, 1.02). Intervention B *vs* control HR=0.86 (95% CI: 0.68, 1.08).
**Participants:** 130 men with metastatic prostate cancer, with disease progression despite androgen deprivation.	**Progression-free survival:** Not reported.
**Intervention A (*n*=44):** Docetaxel (70 mg m^−2^ on day 2 every 21 days)+estramustine (840 mg in three divided doses on days 1–5 and 8–12)+prednisone (10 mg daily) *vs*	**Response rate:** Number of patients responding: Intervention A=nine, intervention B=three, control=one.
**Intervention B (*n*=44):** Docetaxel (35 mg m^−2^ on days 2 and 9 every 21 days)+estramustine (as above)+prednisone (as above) *vs*	**Quality of life response:** Not reported.
**Control (*n*=42):** Mitoxantrone (12 mg m^−2^ on day 1 every 21 days)+prednisone (as above).	**Pain response:** Intervention A *vs* control RR=1.52 (95% CI: 0.74, 3.13). Intervention B *vs* control RR=1.11 (95% CI: 0.50, 2.45).
	**PSA decline:** Intervention A *vs* control RR=4.05 (95% CI: 1.99, 8.21). Intervention B *vs* control RR=3.71 (95% CI: 1.82, 7.58).
	**Grade 3/4 granulocytopaenia:** Intervention A=37%, intervention B=0%, control=48%.
	
**SWOG 9916** Petrylak *et al* (2004)	**Mortality:** HR=0.80 (95% CI: 0.67, 0.97).
**Study design:** Multicentre, stratified open-label RCT.	**Progression-free survival:** HR=1.30 (95% CI: 1.11, 1.52).
**Participants:** 770 men with metastatic prostate cancer, with disease progression despite androgen-ablative therapy and cessation of anti-androgen treatment.	**Response rate:** RR=1.54 (95% CI: 0.74, 3.18).
**Intervention (*n*=386):** Docetaxel (60–70 mg m^−2^ on day 2 every 21 days)+estramustine (three times daily on days 1–5) *vs*	**Quality of life response:** Not reported.
**Control (*n*=384):** Mitoxantrone (12–14 mg m^−2^ on day 1 every 21 days)+prednisone (5 mg twice daily).	**Pain response:** No significant difference (data not provided).
	**PSA decline:** RR=1.85 (95% CI: 1.49, 2.30).
	**Grade 3/4 adverse events:** Intervention=53%, control=33%.
	
Berry *et al* (2002)	**Mortality:** HR=1.13 (95% CI: 0.75, 1.7).
**Study design:** Multicentre, open-label RCT.	**Progression-free survival:** HR=0.64 (95% CI: 0.48, 0.86).
**Participants:** 120 men with asymptomatic prostate cancer that had progressed on at least one hormonal regimen. 86% intervention group and 79% control group had bone metastases, 18% in both groups had lymph metastases.	**Response rate:** RR=1.13 (95% CI: 0.20, 6.24).
**Intervention (*n*=56):** Mitoxantrone (12 mg m^−2^ every 21 days)+prednisone (5 mg orally twice daily) *vs*	**Quality of life response:** Not reported.
**Control (*n*=63):** Prednisone (as above).	**Pain response:** Not reported.
	**PSA decline:** RR=2.03 (95% CI: 1.21, 3.40).
	**Grade 3/4 neutropaenia**: Intervention=48%, control=10%.
	**Grade 3/4 leucopaenia**: Intervention=20%, control=8%.
	
**CCI-NOV22 (Lederle Laboratories)** Tannock *et al* (1996)	**Mortality:** HR=0.91 (95% CI: 0.69, 1.19).
**Study design:** Multicentre, stratified open-label RCT.	**Time to progression:** HR=2.15 (95% CI: 1.46, 3.17).
**Participants:** 161 men with metastatic prostate cancer, with disease progression despite standard hormonal therapy. Patients were required to have symptoms of pain.	**Response rate:** RR=2.33 (95% CI: 1.19, 4.57).
**Intervention (*n*=80):** Mitoxantrone (12 mg m^−2^ every 21 days)+prednisone (5 mg orally twice daily) *vs*	**Quality of life response:** Significant benefits for intervention group compared with control group in terms of duration of improvement for several items.
**Control (*n*=81):** Prednisone (as above).	**Pain response:** Significant benefit for intervention group compared with control group.
	**PSA decline:** RR=1.5 (95% CI: 0.81, 2.79).
	**Grade 3/4 adverse events:** Intervention=22, control=15.
	
**CALGB 9182** Kantoff *et al* (1999)	**Mortality:** HR=1.05 (95% CI: 0.74, 1.49).
**Study design:** Multicentre, stratified open-label RCT.	**Time to progression:** HR=1.50 (95% CI: 1.06, 2.13).
**Participants:** 242 men with metastatic prostate cancer. Antiandrogen withdrawal and disease progression were required before trial entry.	**Response rate:** RR=1.65 (95% CI: 0.56, 4.91).
**Intervention (*n*=119):** Mitoxantrone (14 mg m^−2^ every 21 days)+hydrocortisone (30 mg orally in the morning, 10 mg orally in the evening) *vs*	**Quality of life response:** Significant benefits for intervention group compared with control group for some quality of life items.
**Control (*n*=123):** Hydrocortisone (as above).	**Pain response:** Significant benefit for intervention group compared with control group for pain severity.
	**PSA decline:** RR=1.74 (95% CI: 1.14, 2.66).
	**Grade 3/4 haematopoietic toxicities:** White blood count: Intervention=59%, control=1%. Granulocytes: Intervention=63%, control=1%. Lymphocytes: Intervention=70%, control=15%.
	
Ernst *et al* (2003)	**Mortality:** HR=0.95 (95% CI: 0.71, 1.28).
**Study design:** Multicentre, stratified double-blind RCT.	**Progression-free survival:** HR=1.24 (95% CI: 0.93, 1.64).
**Participants:** 227 men with metastatic prostate cancer, with progressive bone disease despite castrate levels of testosterone. Patients were required to have stable levels of analgesic use for at least 7 days before randomisation.	**Response rate:** RR=1.14 (95% CI: 0.81, 1.59).
**Intervention (*n*=115):** Mitoxantrone (12 mg m^−2^ every 21 days)+prednisone (5 mg twice daily)+clodronate (1500 mg over 3 h every 21 days) *vs*	**Quality of life response:** RR=0.89 (95% CI: 0.64, 1.25).
**Control (*n*=112):** Mitoxantrone (as above)+prednisone (as above)+placebo (1500 mg saline over 3 h every 21 days).	**Pain response:** RR=1.27 (95% CI: 0.83, 1.95)
	**PSA decline:** RR=1.04 (95% CI: 0.68, 1.59).
	**Grade 3/4 granulocytopenia:** Intervention=14, control=14.

CI=confidence interval; HR=hazard ratio; RR=relative risk; RCT=randomised controlled trial.
